# New Three Dimensional-Printed Polyethylene Terephthalate Glycol Liners for Hip Joint Endoprostheses: A Bioactive Platform for Bone Regeneration

**DOI:** 10.3390/ma18061206

**Published:** 2025-03-08

**Authors:** Gheorghe Iosub, Ioana-Alexandra Lungescu, Alexandra Cătălina Bîrcă, Adelina-Gabriela Niculescu, Paul Catalin Balaure, Sorin Constantinescu, Bogdan Mihaiescu, Dragoș Mihai Rădulescu, Alexandru Mihai Grumezescu, Ariana Hudiță, Ionela Andreea Neacșu, Adrian Radu Rădulescu

**Affiliations:** 1 Faculty of Medicine, Carol Davila University of Medicine and Pharmacy, 8 Eroii Sanitari Street, 050474 Bucharest, Romania; iosub.gigi@gmail.com (G.I.); dr.sorin.c@gmail.com (S.C.); radu_radulescu@umfcd.ro (A.R.R.); 2 Department of Science and Engineering of Oxide Materials and Nanomaterials, University Politehnica of Bucharest, 1-7 Gh. Polizu Street, 060042 Bucharest, Romania; alexandralungescu@gmail.com (I.-A.L.); ada_birca@yahoo.com (A.C.B.); adelina.niculescu@upb.ro (A.-G.N.); agrumezescu@upb.ro (A.M.G.); ionela.neacsu@upb.ro (I.A.N.); 3 Research Institute of the University of Bucharest—ICUB, University of Bucharest, 90-92 Panduri, 050663 Bucharest, Romania; bogdanzzz01@gmail.com (B.M.); ariana.hudita@unibuc.ro (A.H.); 4 Organic Chemistry Department, University Politehnica of Bucharest, 1-7 Gh. Polizu Street, 060042 Bucharest, Romania; 5 Department of Biochemistry and Molecular Biology, University of Bucharest, 91-95 Splaiul Independentei Street, 050095 Bucharest, Romania

**Keywords:** three-dimensional printing, PETG porous scaffold, bone tissue engineering, hydroxyapatite (HAp)–chitosan–folic acid immersion-based incorporation

## Abstract

Osteoporosis and bone defects are commonly observed in postmenopausal women, often linked to decreased folic acid levels, which play a crucial role in bone metabolism and regeneration. This study investigates 3D-printed polyethylene terephthalate glycol (PETG)-based porous scaffolds impregnated with chitosan (CS), hydroxyapatite (HAp), and folic acid (FA) for bone tissue engineering applications. The PETG-CS scaffold serves as the primary structural framework, with HAp incorporated to enhance bioactivity through its osteoconductive and osteoinductive properties. FA was included to address potential deficiencies in bone quality and to stimulate cellular differentiation. The scaffolds were fabricated using precise 3D printing techniques, yielding structures with controlled porosity. Physicochemical analyses confirmed the successful integration of HAp and FA into the PETG-CS matrix. Biological evaluations using preosteoblast cell lines demonstrated enhanced cell viability, proliferation, and biocompatibility of the scaffolds. These findings highlight the promising applications of PETG-CS-HAp-FA scaffolds in bone tissue engineering, providing a platform for future investigations into personalized regenerative therapies.

## 1. Introduction

According to Atala and Lanza [1], tissue engineering is “an interdisciplinary field of research which applies the principles of engineering and life sciences towards the development of biological substitutes that aim to maintain, restore or improve tissue function”. Although bone is a resilient tissue with a self-healing capacity, it is not able to repair critical-size defects caused by various conditions, such as trauma, infections, osteoporosis, aging, and cancer [2,3,4]. To treat such conditions, classical reconstructive orthopedic surgery uses bone grafting, including autologous bone transplantation, allogenic bone transplantation, xenograft bone transplantation, and artificial bone transplantation [2]. All these techniques exhibit severe limitations and drawbacks. While immunological graft rejection remains a barrier to safe allogenic and artificial bone transplantations, autologous bone transplantation presents important drawbacks related to a limited autologous bone supply, to graft donor site morbidity, and to the risk of infections at both the host and donor graft sites, resulting in low therapeutic effectiveness and even treatment failure [5].

Bone tissue engineering (BTE) aims to overcome the aforementioned issues and to mitigate complications associated with conventional bone grafting procedures [6,7,8]. Prevalent elements involved in BTE are scaffolds, cells, cytokines, and growth factors [7].

Scaffolds represent 3D structures mimicking the architecture and properties of the extracellular matrix (ECM) and providing a suitable platform for cells’ adhesion, growth, and differentiation [9,10,11,12,13]. BTE scaffolds must fulfil a series of essential intricate biological requirements. An ideal BTE scaffold should be characterized by good biocompatibility, favorable bioactivity in terms of osteoconductive, osteoinductive, and osteointegration capacities, and proper biodegradability at a rate matching the rate of tissue regeneration since BTE scaffolds are intended to provide temporary mechanical integrity at the defect site until the engineered tissue ingrowth results in complete tissue repair and regaining of the normal biomechanical functionality [14,15]. Osteoconductivity refers to the property of a material to function as a scaffold for bone formation, meaning that the material provides a suitable porous 3D substrate with an interconnected network of pores, channels, and pipes supporting cell growth and migration, the diffusion of oxygen, nutrient flow, clearance of waste products, vascularization, and better spatial organization of growing cells [9,14,16,17,18,19,20]. Osteoinduction can be viewed as the acceleration of the regenerative process by means of chemical signals stimulating the differentiation of undifferentiated perivascular mesenchymal cells to osteoblasts or, in other words, new bone tissue development [16]. There is a delicate interplay between the scaffold properties related to mechanical and biological factors [2]. For instance, the increased porosity of the scaffold is positively correlated with biological activity, including cell growth and the transport and distribution of nutrients, but leads to decreased mechanical strength. Similarly, an increased surface area aids the initial adhesion of cells to the surface of the scaffold but, on the other hand, it leads to a faster degradation of scaffolds. Although changes in the elastic modulus render reinforced mechanical properties to the scaffold material, they simultaneously alter tissue growth rate and type [21]. It appears to be an extremely challenging task for any material to fully meet all these requirements. Calcium-based biomaterials, such as hydroxyapatite (HA), which is the predominant inorganic component found in human bones and tricalcium phosphate (TCP in both α and β crystalline phases), as well as ceramic materials, like whitlockite (WH), a calcium phosphate including a magnesium ion, showed good bioactive properties [22,23,24,25,26,27]. Polyethylene terephthalate (PET) has been extensively utilized in the development of innovative materials for bone tissue regeneration applications over the past few decades [28,29]. Its suitability for these applications stems from its unique properties, which have garnered approval from the China Food and Drug Administration. One notable example of PET’s application is the Ligament Advanced Reinforcement System (LARS), a revolutionary solution designed to address Anterior Cruciate Ligament (ACL) ruptures. ACL injuries are a common affliction caused by both professional sports and recreational activities, often necessitating reconstructive surgery. The LARS system has been successfully employed to provide a durable and reliable repair option for individuals suffering from this debilitating condition [30,31,32]. Beyond its application in LARS, PET has been explored as a versatile material for various orthopedic and maxillofacial applications. For instance, it has been used as a membrane for mandibular reconstruction, providing a scaffold for bone growth and regeneration. Additionally, PET has been utilized as a patch for repairing rotator cuff tears, a common injury affecting the shoulder joint. Its use has also been extended to other forms, such as screws, plates, and implants, demonstrating its adaptability and potential in the field of orthopedic and maxillofacial surgery [33,34,35,36,37]. Such biocomposites with improved functionalities include co-polymers, like poly (lactide-*co*-glycolide) (PLGA), polymer–polymer blends, and ceramic (for instance HA, bioglass) biopolymers (collagen, gelatin, chitosan, and alginate), or synthetic biocompatible and bioresorbable polymers (PLGA, poly(lactic acid), and PLA) composites [38,39,40,41,42,43].

There are several fabrication methods to produce scaffolds for tissue engineering from which we list: the gas injection foaming method [44], basic electrospinning or in combination with additive manufacturing [45,46,47], freeze drying [48,49], and various 3D printing methods.

Additive manufacturing (AM), often referred to as 3D printing, has fundamentally transformed the procedure of fabricating scaffolds in the realm of biomedical engineering. This technique allows for the development of scaffolds with precise architecture that closely resemble actual tissues. AM is a digital molding technology working on the principle of layered manufacturing and layer by layer superposition. The 3D model of the repair site, first obtained using CT scanning or magnetic resonance imaging, is further sliced with the aid of computer-aided design (CAD) software to obtain the data of each layer, which are eventually imported into the 3D printing system [2]. Using these data, the printing apparatus prepares the bone scaffold by additive superposition layer by layer of the materials, according to the layered data. This enables the production of customized implants, which is particularly advantageous in the field of personalized medicine since it allows for the creation of scaffolds that are specifically tailored to meet a patient’s unique anatomy or damage. Advanced technologies, like fused deposition modeling (FDM) [50,51], selective laser sintering (SLS) [52,53,54], and stereolithography (SLA), enable the construction of scaffolds with high precision and the intricate adjustment of inside pores size and architecture. Extrusion bioprinting is highly compatible with the inclusion of thermally sensitive materials like cells and bioactive substances into the bioink composition, since the printing technique is does not involve a heating process [55]. Moreover, the application of additive manufacturing techniques in the creation of dynamic scaffolds receives significant enhancement through the development of 4D printing, which incorporates time as the fourth dimension. Building on the principles of 3D printing, 4D printing employs smart materials that allow the printed scaffold to change its shape and size over time in response to specific locally applied stimuli or alterations in the microenvironment [56].

However, in addition to their innovative potential, the engineered solutions must also be economically viable, biodegradable, and minimize adverse reactions, while delivering reliable and sustainable results both in the short and long terms. This study aims to develop a porous PETG scaffold using 3D printing technology, a technique that offers flexibility in terms of cost and scalability. Additionally, the current research incorporates a secondary objective: valorizing food waste to obtain hydroxyapatite, a crucial component for the material. Furthermore, folic acid has been incorporated into the formulation to address the specific needs of women experiencing menopause, which is often accompanied by osteoporosis and hormonal imbalances [57,58,59,60]. To enhance the eco-friendliness of the material and promote a favorable body response, the active powders are embedded in a chitosan matrix. This biocompatible carrier provides structural support, coverage, and facilitates the diffusion of hydroxyapatite and folic acid through the porous PETG scaffold. In the present study, we report on the fabrication, physicochemical characterization, and in vitro biological evaluation of BTE scaffolds based on a polyethylene terephthalate glycol (PETG) matrix incorporating chitosan (CS), hydroxyapatite (HAp), and folic acid (FA).

## 2. Materials and Methods

### 2.1. Materials

To fabricate the porous cubes, a polyethylene terephthalate glycol (PETG) filament from Norditech (SC NORDITECH MACHINERY SRL, Maramureș, Romania) was utilized. Hydroxyapatite was synthesized using a natural calcium carbonate precursor derived from the soft membrane of eggshells, which were sourced from locally purchased eggs. The synthesis process also incorporated various chemical reagents, including hydrochloric acid (HCl), hydrogen peroxide (H_2_O_2_), diammonium phosphate ((NH_4_)_2_HPO_4_), and an ammonium hydroxide solution (NH_4_OH). Additionally, medium-molecular-weight chitosan (MMWC) and sodium hydroxide (NaOH) were used to prepare a neutral pH chitosan stock solution. Folic acid (C_19_H_18_N_7_NaO_6_) was also used as a material in this study. All reagents were analytical grade and obtained from Sigma-Aldrich/Merck (Darmstadt, Germany), without further purification.

### 2.2. Methods

#### 2.2.1. Scaffold Fabrication and the Synthesis of Biocomposite Scaffold Materials

Scaffold manufacturing using 3D printing:

The PETG scaffolds were 3D printed using the Sovol SV04 IDEX Dual 3D printer (Sovol, Shenzhen, China). The printing medium utilized was the Norditech PET-G filament (SC NORDITECH MACHINERY SRL, Maramureș, Romania), a premium commercial PETG material known for its exceptional durability and longevity. The printing process employed a nozzle diameter of 0.4 mm, ensuring precise layer deposition and intricate detail. The layer height was adjusted to 0.2 mm, striking a balance between print quality and processing speed. This adjustment facilitated the formation of seamless layers and a well-defined framework for the scaffolds.

The printing temperature was maintained at 235 °C, the optimal setting for PETG, which ensured a smooth filament flow and strong interlayer adhesion. To prevent distortion during printing, the print bed temperature was elevated to 75 °C, guaranteeing that the scaffolds maintained their flatness and dimensional accuracy. The printing speed was optimized to 60 mm/s, facilitating efficient production while preserving the high quality of the final output. Each PETG scaffold consisted of 50 layers and required approximately 7 min (per each cube) to complete the printing process.

To ensure a robust and reliable structure, the infill density was set to 100%, providing optimal density and mechanical strength for the scaffolds. Ultimately, a total of nine cubic pieces of PETG scaffolds, each measuring 1 × 1 × 1 cm, were successfully produced without any post-treatment. The scaffolds demonstrated uniform dimensions and a sturdy structure, making them ideal candidates for further investigation and application.

Synthesis of Hydroxyapatite (HAp):

For HAp preparation, a green synthesis approach was adopted, utilizing eggshells as a natural source of calcium carbonate. A batch in the range of 30–40 eggshells was worked up by carefully getting rid of the eggshell membrane, a protein-based tissue found between the hard eggshell and the egg white. To this end, the eggshells were placed for 10 min in a Berzelius beaker containing 2 L of 0.5 M aqueous hydrochloric acid under magnetic stirring. Afterwards, the eggshell membranes were removed with the aid of a tweezer. The mineral component was further cleaned using a H_2_O_2_-assisted ultrasonic bath treatment for 30 min. Following the ultrasound treatment, the eggshells underwent a thorough rinse with distilled water before being carefully dried in an oven for 48 h at a temperature of 60°C. Once dried, the materials were finely ground for 15 min using a mechanical grinder and then separated by granulation using a sieve with a mesh size of 250 μm. Following the grinding process, the powder underwent calcination at a temperature of 1000 °C for 5 h, with a gradual heating rate of 5 degrees per minute. The resulted powder was suspended in 200 mL of distilled water to which 200 mL of aqueous diammonium phosphate solution was added dropwise under magnetic stirring. The pH of the reaction mixture was adjusted to a range of 10–10.5 by dropping ammonium hydroxide solution 25 wt.%. Next, the mixture was poured into the containers of microwave-assisted equipment for hydrothermal maturation. The synthWAVE equipment (Milestone Srl, Sorisole, Bergamo, Italy) was operated as follows: the vials tightly sealed with Teflon caps were positioned on a rotating holder placed in a water bath, which allowed proper energy and heat transfer. The temperature in the reaction chamber was precisely maintained at 120 °C for 12 min while a pressure of 2.5 bar was applied thereby ensuring that the solutions remained stable, preventing any unwanted boiling. After the completion of the microwave field process, a cooling device that was integrated into the system quickly reduced the temperature. Eventually, the solid formed was separated by filtration rinsed with distilled water to lower the pH from 10 to a normal range of 7–7.5, and air-dried overnight [61].

Preparation of CS, HAp-CS, and HAp-CS-FA stock:

A 2% (*w*/*v*) CS stock solution was prepared in a low acidic medium of acetic acid. A 1.5 M NaOH solution was gradually added until the pH shifted from an initial acidic value of 4 to a slightly basic physiological pH in the range of 7.2–7.4.

A HAp stock was prepared by the thorough dispersion of HAp powder in 10 mL distilled water using an ultrasonic bath. Next, the prepared HAp stock was combined with 100 mL of CS stock and further sonicated to obtain the CS-HAp samples.

Once the mixture achieved homogeneity, it was equally divided in two parts of 50 mL each. One part was further modified by the addition of 1.5 g of FA powder and ultrasonicated until complete powder dispersion to eventually obtain the CS-HAp-FA solution.

Modified PETG scaffolds were produced by immersing three specimens of previously 3D-printed PETG scaffolds in 50 mL of each solution—chitosan (CS), chitosan–hydroxyapatite (CS-HAp), and chitosan–hydroxyapatite–folic acid (CS-HA-FA)—for 24 h to ensure the impregnation of the solutions throughout the entire scaffold structure. After this period, the soaked scaffolds were air-dried at room temperature.

#### 2.2.2. Physicochemical Characterization Techniques

X-ray diffraction (XRD) analysis was conducted using a PANalytical Empyrean (PANalytical, Almelo, The Netherlands) instrument configured in Bragg–Brentano geometry. The system featured a Cu anode X-ray tube (λCuKα = 1.541874 Å) with a line focus, a programmable divergent slit on the incident side, and a programmable anti-scatter slit mounted on the PIXcel3D detector on the diffracted side. The data were collected over a 2θ angle range of 15–80° for each sample, with a step size of 0.02° and an acquisition time of 100 s per step.

Scanning electron microscopy (SEM) coupled with energy dispersive X-ray spectroscopy (EDS) was employed to examine the morphological and topological features, as well as the elemental composition, of the synthesized HAp powder samples and 3D-printed scaffolds. The powder samples were mounted on a carbon-seeded slide and analyzed using an Inspect F50 scanning electron microscope (Thermo Fisher—FEI, Eindhoven, The Netherlands). Images were obtained by capturing the secondary electron beam generated by electron scattering, using an accelerating voltage of 30 keV.

Fourier Transform Infrared Spectroscopy (FTIR) was conducted to assess the compositional integrity of the prepared samples. Spectral analysis was performed using a Nicolet 6700 FT-IR spectrometer (Thermo Fischer Scientific, Madison, WI, USA) equipped with a ZnSe crystal. Measurements were carried out at room temperature, with each sample subjected to 32 scans in the spectral range of 4000–1000 cm^−1^, at a resolution of 4 cm^−1^. Data acquisition and processing were performed using OmnicPicta software (version 8.2, Thermo Fischer Scientific, Madison, WI, USA).

Raman confocal microscopy was performed using an inVia Qontor system (Renishaw, Torino, Italy) equipped with a 785 nm laser and a 20× objective lens.

#### 2.2.3. Assessment of Biocompatibility Using In Vitro Testing

To evaluate the biocompatibility of porous cubes made from PETG, both unmodified and modified with CS, HAp, and folic acid (FA), the human preosteoblast cell line hFOB was used as an in vitro cellular model. The cells were cultured in a medium recommended by the manufacturer, consisting of a 1:1 mixture of Ham’s F12 and Dulbecco’s Modified Eagle’s Medium (DMEM), supplemented with 2.5 mM of L-glutamine (without phenol red), 0.3 mg/mL of G418, and 10% fetal bovine serum (FBS). The cells were maintained in sterile flasks under optimal growth conditions at 37 °C and 5% CO_2_. Prior to cell seeding, the 3D-bioprinted cubes were sterilized by exposing them to UV radiation for 10 min on each side. Following sterilization, the cells were detached from the culture surface using trypsin/EDTA and quantified with a hemocytometer. Each 3D-printed cube was coated with 0.5 × 10^4^ cells applied as a 25 µL droplet. After a one-hour seeding period, the 3D bioprinted cubes were submerged in an appropriate culture medium and placed in an incubator for 24 h at 34°C with 5% CO_2_. After this incubation period, the following parameters were assessed: (i) cell viability was measured using the quantitative MTT assay; (ii) the distribution of preosteoblasts within the structure of the 3D porous cubes, along with cell viability, was evaluated by the Live/Dead test; (iii) the cytotoxicity of the 3D porous cubes was determined by measuring the level of lactate dehydrogenase (LDH) released into the culture medium; and (iv) nitric oxide (NO) production was quantified using the Griess assay.

##### MTT Assay

To evaluate cell viability and proliferation, the tetrazolium dye 3-(4,5-dimethylthiazol-2-yl)-2,5-diphenyltetrazolium bromide (MTT) assay was conducted using the MTT reagent from Sigma Aldrich (St. Louis, MO, USA), following the protocol outlined below: At the specified experimental intervals, the culture medium was carefully removed from the cell monolayers and replaced with an MTT solution (1 mg/mL), which had been prepared in advance by dissolving MTT in DMEM without FBS. The samples were then incubated at 37 °C for four hours. After incubation, the MTT solution was removed from the cell monolayers, and the resulting formazan crystals were dissolved in DMSO. The absorbance of the resulting solution was measured in triplicate for each sample at a wavelength of 550 nm using the FlexStation III multimodal plate reader (Molecular Devices, San Jose, CA, USA). The data were analyzed using GraphPad Prism 6 software, applying the ANOVA statistical test with the Bonferroni correction. The results are presented as the mean of three biological replicates, along with the standard deviation, and are expressed as the percentage cell viability relative to the control sample (PETG/CS), which was considered as having 100% viability. A significance threshold of *p* < 0.05 was established to determine the statistical significance.

##### Live/Dead Test

To assess cell health, the Live/Dead test was performed using the Live/Dead Viability Cytotoxicity Kit for mammalian cells (Invitrogen^TM^, Waltham, MA, USA). The following protocol was implemented: Before starting the experiment, the Live/Dead staining solution was prepared according to the kit instructions, which involved diluting the components in a serum-free medium to achieve final concentrations of 2 μM calcein AM and 4 μM ethidium bromide (BrEth). The culture medium was removed from the culture vessels, and the cell monolayers were washed with PBS buffer to label the samples. After washing, the samples were incubated in the prepared Live/Dead solution for 15 min at room temperature, protected from the light. The volume of the Live/Dead solution was adjusted to ensure complete coverage of the cell monolayer, tailored to the size of the culture area. Once the incubation period was complete, the samples were examined using fluorescence on the Olympus IX73 inverted fluorescence microscope (Olympus Life Science, Waltham, MA, USA). The resulting images were then captured and processed using CellSense Imaging Software v8.0.2.

##### LDH Assay

The colorimetric LDH test is a quantitative method used to evaluate cytotoxicity by measuring the enzymatic activity of lactate dehydrogenase (LDH) released into the culture medium from cells with compromised membranes through spectrophotometry. This method is based on the conversion of NAD^+^ to NADH and H^+^ by LDH. NADH and H^+^ reduce a tetrazolium salt into a soluble formazan derivative that exhibits a distinct color. The intensity of the red color of formazan can be measured spectrophotometrically at a wavelength of 490 nm. When the plasma membrane is damaged, cell viability diminishes, leading to the release of LDH into the culture medium. To assess cytotoxicity using the LDH test, we employed the In Vitro Toxicology Assay Kit Lactic Dehydrogenase-based TOX7 from Sigma Aldrich. This kit comprises three essential components: lactate substrate, dye, and cofactor NAD+. The protocol for conducting the test is as follows: At designated experimental intervals, 100 μL of the culture medium was extracted from each sample under investigation. The collected samples were then transferred into a 96-well plate and mixed with 50 μL of lactate substrate, 50 μL of dye solution, and 200 μL of cofactor solution, as per the manufacturer’s protocol. The 96-well plate was allowed to incubate for 30 min at room temperature in a dimly lit area. After the incubation period, the reaction was quenched by carefully adding 30 μL of 1N HCl to each well. d. The optical density (OD) of the solution was measured for each sample in triplicate using the FlexStation III multimodal plate reader (Molecular Devices) at 490 nm. The data were analyzed using GraphPad Prism 6 software, employing the ANOVA statistical test with the Bonferroni correction. The results are presented as the mean of three biological replicates along with the standard deviation, with a significance threshold of *p* < 0.05 established to determine statistical significance.

##### Griess Test

The Griess method is employed to measure the production of nitric oxide (NO) by quantifying the amount of nitrite (NO_2_^−^) released into the culture medium. Under specific conditions, a red azo dye complex forms when nitrite reacts with sulfanilamide and the NED reagent in a weakly acidic medium (phosphoric acid). The intensity of the red color of the azo dye complex can be measured spectrophotometrically at wavelengths ranging from 520 to 550 nm. The amount of nitrite released into the culture medium is directly correlated with the amount of NO produced by the cells. To measure nitric oxide production, we used the Griess reagent from Promega, which consists of two components: sulfanilamide and the NED reagent (N-1-naphthylethylenediamine dihydrochloride). The protocol for using this reagent is as follows: At specified experimental intervals, a volume of 50 μL of culture medium was extracted from each sample under study. The collected media were then carefully transferred into a 96-well plate. Using the 0.1 N nitrite standard provided in the kit, various dilutions were prepared and pipetted into a separate 96-well plate. These dilutions included concentrations of 100, 50, 25, 12.5, 6.25, 3.13, and 1.56 μM, which allowed us to create a standard curve for the experiment. In each well containing the culture medium, 50 μL of the sulfanilamide solution was added. The 96-well plate was then incubated for 10 min at room temperature in the dark, with the same procedure applied to the plate used for creating the standard curve. After this incubation period, 50 μL of the NED reagent from the kit was added to each well. The plate was incubated for an additional 10 min at room temperature in the dark, following the same procedure for the standard curve. The optical density (OD) of the solution was measured in triplicate for each sample using the FlexStation III multimodal plate reader (Molecular Devices) at a wavelength of 530 nm. The data were analyzed using GraphPad Prism 6 software, employing the ANOVA statistical test with the Bonferroni correction. To construct the standard curve, we used the concentrations linked to their respective absorbance values, subsequently extrapolating the absorbance values of the samples onto the obtained curve. The results are presented as the average of three biological replicates, along with the standard deviation, with a *p*-value of less than 0.05 considered statistically significant.

## 3. Results

### 3.1. Phisicochemical and Morphological Characteristics of the HAp Powder and of the Fabricated PETG-Based Scaffolds

**Figure 1:**
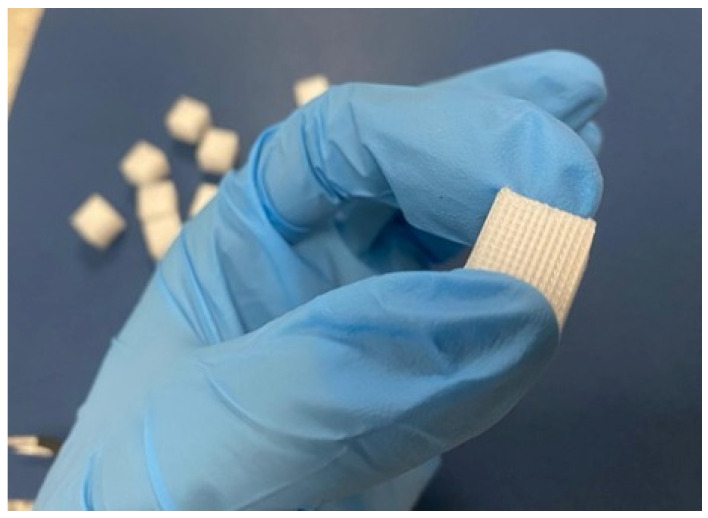
Three-dimensionally printed scaffold.

Within the examined range of 2θ, nine distinct diffraction interference patterns were observed at estimated angles of 10°, 25°, 32°, 33°, 35°, 40°, 46°, 50°, and 54° (Figure 1). The tabulated data provided by the ASTM sheets indicate that the diffraction maxima observed in the experimentally synthesized powders correspond to the diffraction planes (0 0 2), (2 1 1), (1 1 2), and (3 0 0) of hydroxyapatite, which crystallizes in a hexagonal system. The diffractogram reveals a small full width at half maximum, suggesting that the crystallite size is large. This finding is further supported by calculations based on the Debye–Scherrer equation, which indicates that the average crystallite size is 69.55 Å, as determined from the highest intensity maximum corresponding to the (2 1 1) diffraction plane.

**Figure 2:**
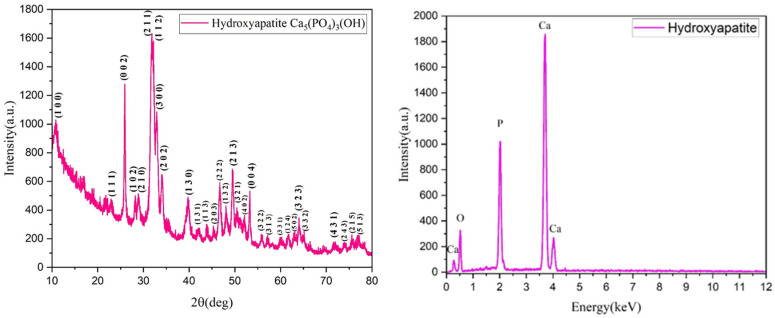
X-ray diffraction pattern and EDS spectrum obtained for the green synthesized Hap powder.

The EDS spectrum (Figure 2) of the HAp powder revealed prominent intensities of the elements of calcium, oxygen, and phosphorus, the chemical elements present in hydroxyapatite. Furthermore, it is noteworthy that the calcium element exhibits the highest intensity peak, as it is present in the greatest amount within the structure of calcium phosphates.

**Figure 3:**
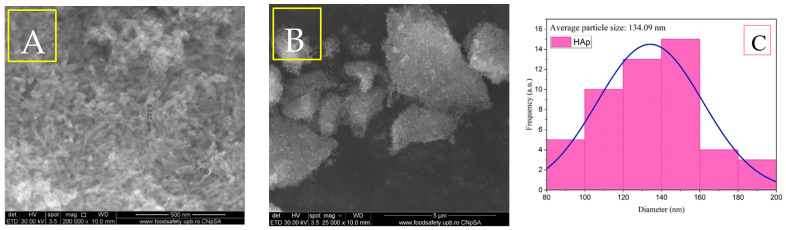
SEM micrographs (**A**,**B**) obtained for Hap powder and the particle size distribution presented as a histogram (**C**).

Figure 3 presents micrographs obtained from the analysis of hydroxyapatite powder using a scanning electron microscope. In image A, at a magnification of 200,000×, the distinct morphologies of hydroxyapatite are visible, arranged in clusters of luminous, elongated particles that are uniformly distributed. The particle size ranges from 80 to 200 nm, as further illustrated by the histogram in Image C, which shows a unimodal distribution with an average particle diameter of 134.09 nm. In Image B, at a lower magnification (25,000×), very fine particles predominantly grouped into clusters reflect the natural organization of HAp, indicating its characteristic aggregation pattern.

**Figure 4:**
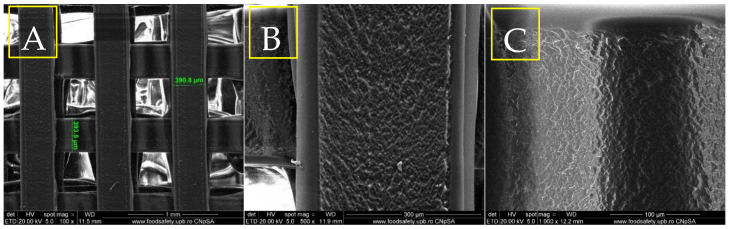
SEM micrographs of PETG at different magnifications, 100× (**A**), 500× (**B**), 1000× (**C**).

**Figure 5:**
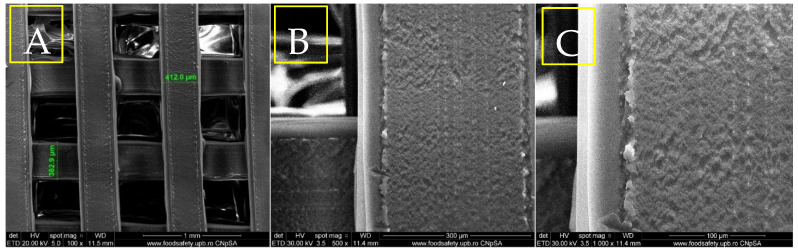
SEM micrographs of PETG/CS at different magnifications, 100× (**A**), 500× (**B**), 1000× (**C**).

**Figure 6:**
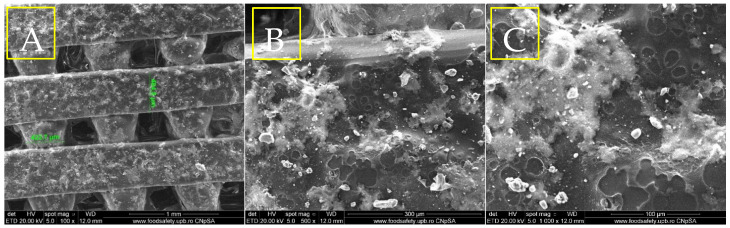
SEM micrographs of PETG/CS-Hap at different magnifications, 100× (**A**), 500× (**B**), 1000× (**C**).

**Figure 7:**
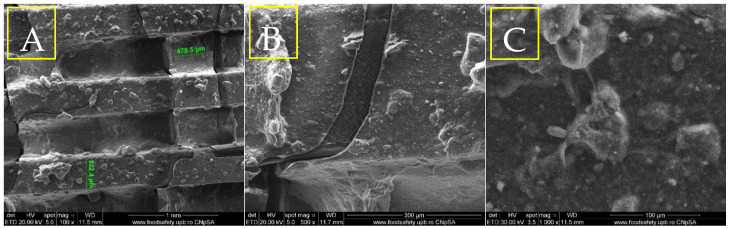
SEM micrographs of PETG/CS-Hap-FA at different magnifications, 100× (**A**), 500× (**B**), 1000× (**C**).

The results of the physicochemical analyses conducted on the porous PETG scaffolds are presented below. Given that PETG typically requires coating with HAp and bioactive substances for bone tissue applications, the initial evaluation focuses on SEM analysis (Figure 4, Figure 5, Figure 6 and Figure 7). This assessment examines the morphostructure of the pores and PETG layers following successive impregnation with CS, CS-HAp, and CS-HAp-FA.

The scanning electron microscopy image in Figure 4A, captured at a magnification of 100×, reveals a large-scale grid formation within the structure, along with uniform pore sizes of approximately 390 µm. This design facilitates effective cell infiltration and nutrient exchange, which are vital for bone tissue creation and blood vessel formation. The systematic organization of the pores underscores the precision of the 3D printing process, essential for ensuring the structural integrity and mechanical properties that mimic the natural composition of bone. SEM image 4B, taken at a larger magnification (500×), provides a closer examination of the filament’s surface morphology. The surface exhibits a rough texture with discernible micro-textures, which can enhance cellular adhesion and growth. These surface characteristics are critical for promoting osteointegration, as they increase the surface area available for bone cells to attach and generate new tissue. The presence of micro-roughness and the uneven surface of the three-dimensional structure, as observed at 1000× magnification (Figure 4C), are crucial for enhancing the connections between cells and the surface. The irregular surface structure of the PETG scaffold suggests its potential to provide an optimal environment for bone cell development, facilitating both structural stability and biological function in the bone healing process.

The first scanning electron microscopy image in Figure 5A (100× magnification) reveals that the PETG–chitosan scaffold displays a highly organized grid pattern characterized by uniform, rectangular pores. This observation indicates that the 3D printing process was precise and meticulously controlled. The pores have diameters ranging from 382 µm to 412 µm and are consistently distributed, creating a uniform pattern throughout the scaffold. The edges of the grid appear well-defined and unchanged, suggesting that parameters such as the extrusion temperature and speed were optimized to ensure a seamless flow of material and minimize deformation during solidification. Additionally, the surfaces of the filaments appear smooth under this magnification, with no visible flaws or abnormalities. Figure 5B (500× magnification) offers a closer view of the scaffold’s filament, revealing a more intricate surface texture. The surface exhibits a micro-rough texture, characterized by small, granular features that may be attributed to the presence of chitosan. These variations likely result from the blending of materials or additional treatments applied after printing. Additionally, the observed differences may indicate slight irregularities during the cooling or solidification processes of the extruded material. The edges of the filament remain smooth and continuous, showing no noticeable signs of warping or material buildup. Figure 5C, at 1000× magnification, provides an even more detailed perspective of the scaffold, highlighting a pronounced surface roughness at a smaller scale. The fine texture on the filament suggests that the material mixture, particularly the PETG–chitosan blend, may contain small compositional variations, resulting in the observed micro-roughness. The uniformity of this roughness across the surface indicates that it is an intrinsic characteristic of the material rather than a defect in the printing process. Despite the higher magnification, the scaffold structure remains intact, and the edges of the filament show no signs of damage.

The micrograph (Figure 6A), taken at a magnification of 100×, reveals a distinct and consistent porous structure in the PETG/CS—HAp scaffold. The pores measure approximately 432 µm in width, indicating a methodical and uniform printing process. The scaffold’s surface is textured and features noticeable deposits dispersed throughout the layers, likely resulting from the integration of HAp. This mineral component enhances the granular appearance of the surface, while the scaffold layers appear seamlessly fused, maintaining their structural integrity.

The second SEM image in Figure 6B, captured at a magnification of 500×, provides a more detailed view of the scaffold’s surface. It reveals a combination of smooth and uneven regions, with clearly defined clusters of HAp particles embedded within the scaffold material. The hydroxyapatite appears as granular or crystalline structures irregularly distributed across the surface, suggesting variability in the blending process during scaffold fabrication. The third SEM image (Figure 6C), taken at a magnification of 1000×, displays closely interconnected HAp particles, which appear as small, irregular crystalline formations contrasting with the smoother PETG/CS background. The hydroxyapatite tends to aggregate into clusters or agglomerates, both embedded within the scaffold matrix and extending beyond the surface. At this high magnification, the scaffold surface reveals micro-scale voids or indentations, likely formed by the distribution of the HAp particles.

**Figure 8:**
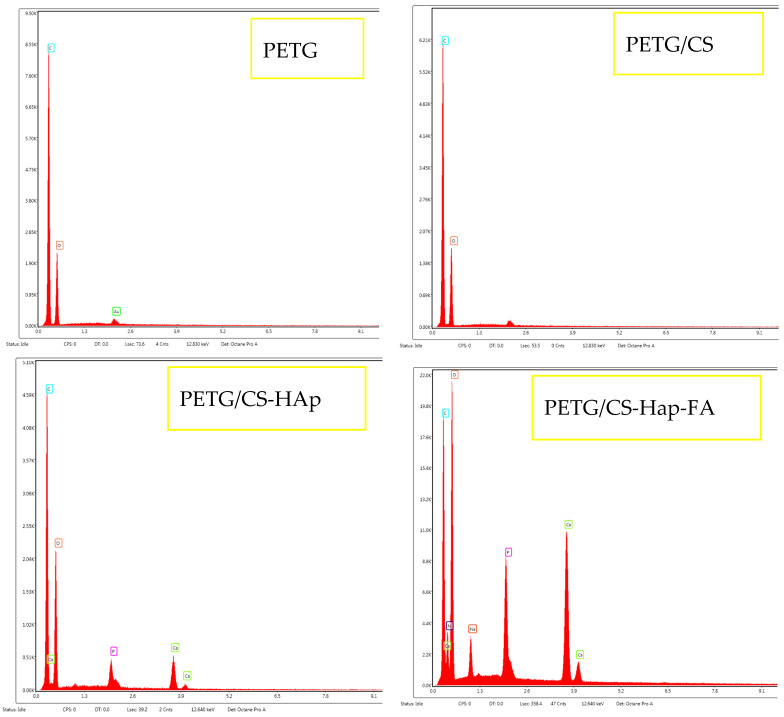
EDS spectra for PETG, PETG/CS, PETG/CS-Hap, and PETG/CS-Hap-FA.

From a morphological perspective, the incorporation of folic acid induces subtle yet meaningful modifications to the surface characteristics of the porous PETG scaffolds. Microscopy images of the PETG/CS-HAp-FA (Figure 8) sample reveal a smoother and more uniform surface, particularly in areas where the chitosan-based bioactive mix has impregnated the pores and coated the scaffold surface. A key observation is the enhanced homogeneity in the distribution of hydroxyapatite particles. Unlike the sample without folic acid, where particle agglomerations were evident, the HAp particles in the folic acid-containing scaffold appear more evenly dispersed throughout the structure. This suggests that folic acid improves the dispersion and stabilization of hydroxyapatite within the chitosan matrix, preventing the formation of undesired clusters. Additionally, the presence of folic acid contributes to a thicker and denser “covering layer”, ensuring a more effective impregnation across the entire porous architecture of the scaffold. This more cohesive and integrated coating suggests an enhanced interaction between folic acid, chitosan, and hydroxyapatite, leading to a more stable and uniform bioactive layer. Overall, these findings indicate that folic acid enhances the properties of the chitosan-based composite, improving its ability to adhere to and infiltrate both the pores and the surfaces of the PETG scaffolds. This modification may positively influence the scaffold’s bioactivity and potential performance in bone tissue engineering applications.

The EDS spectra reveal the elemental composition of each analyzed sample. In the PETG and PETG/CS samples, only carbon and oxygen are detected. However, with the incorporation of HAp and FA, additional elements appear, calcium and phosphorus, confirming the presence of HAp, while nitrogen and sodium are associated with FA, indicating the successful integration of the bioactive substances used in this study.

**Figure 9:**
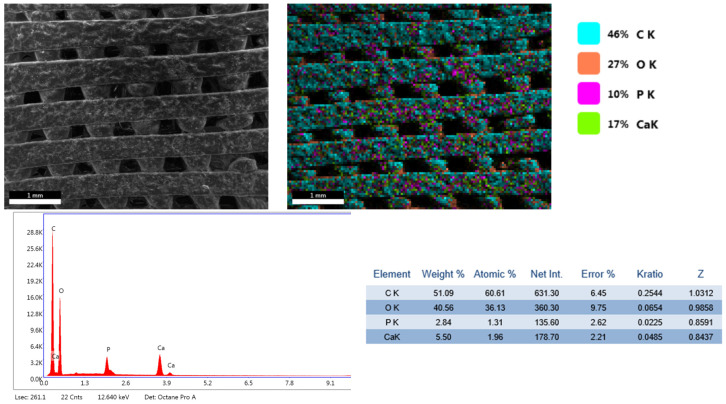
EDS elemental mapping performed for PETG/CS-Hap.

**Figure 10:**
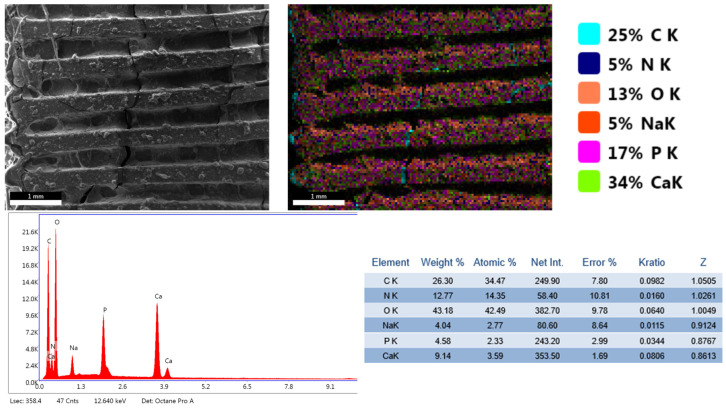
EDS elemental mapping performed for PETG/CS-Hap-FA.

Furthermore, the PETG/CS-HAp and PETG/CS-HAp-FA samples underwent elemental mapping analysis (Figure 9 and Figure 10) to assess the distribution of key elements associated with HAp and folic acid. The mapping results confirm the presence of calcium and phosphorus, which are characteristic of HAp, as well as nitrogen and sodium, indicative of FA incorporation. These findings demonstrate the successful integration of both bioactive components within the porous scaffold structure, ensuring their distribution across the surface and within the internal porous network. This further supports the potential bioactivity and functionality of the scaffolds for bone tissue engineering applications.

**Figure 11:**
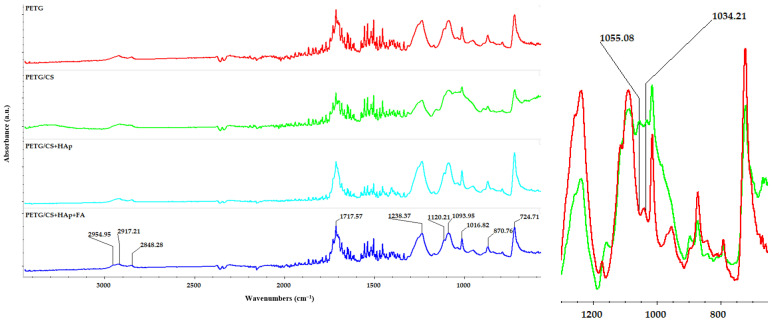
FTIR spectra for PETG, PETG/CS, PETG/CS-Hap, and PETG/CS-Hap-FA, and detailed FT-IR spectra of PETG and PETG/CS samples.

Another analysis conducted on the porous PETG/CS scaffolds was FTIR spectroscopy. Figure 11 presents the FTIR spectra obtained for the analyzed samples.

The FTIR spectrum of PETG confirms the presence of key functional groups associated with its polyester structure. A strong C=O stretching band at ~1717 cm^−1^ indicates the presence of ester (-COO-) bonds, while peaks in the 1600–1500 cm^−1^ region correspond to C=C stretching vibrations of the aromatic benzene rings. Aliphatic C-H stretching bands appear at 2954, 2917, and 2848 cm^−1^, confirming the presence of CH_2_ and CH_3_ groups in the polymer backbone. Additionally, C-O stretching at 1238 cm^−1^ and C-H bending at 870 cm^−1^ further validate the polymer’s ester and aromatic ring structures.

Compared to standard PET, PET-G exhibits broader absorption bands due to its lower crystallinity, which results from glycol modification. The C=O stretching at 1717 cm^−1^ appears slightly broader. The fingerprint region (1400–600 cm^−1^) reveals C-O stretching (1116–1090 cm^−1^) and aliphatic C-H bending, which reflect PET-G’s structural flexibility (Figure 11).

The FTIR spectrum of the PET-G/CS sample exhibits a new absorption band at 1055 cm^−1^, which is absent in the PET-G spectrum (Figure 11—detailed), indicating its origin from chitosan. This peak corresponds to C-O stretching vibrations characteristic of the polysaccharide backbone in chitosan, confirming its presence in the modified polymer matrix. In contrast, the 1034 cm^−1^ peak, observed in both spectra, remains unshifted.

**Figure 12:**
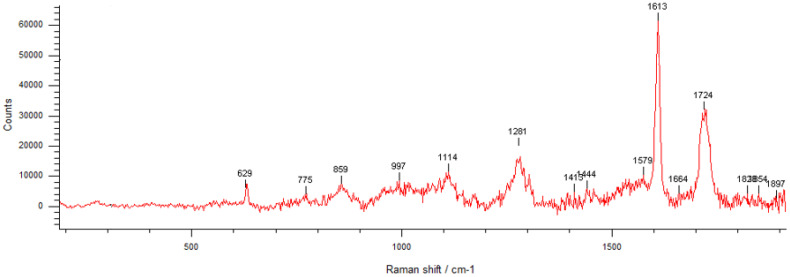
Raman spectrum of PETG/CS/Hap-FA.

The Raman spectrum of PETG/CS/Hap-FA (Figure 12) exhibits characteristic vibrational bands corresponding to its polyester structure, including ester (-COO-), aromatic (C=C), and aliphatic (C-H) functional groups. The strong peak at 1724 cm^−1^ is attributed to C=O stretching, confirming the presence of ester bonds, while the 1613 cm^−1^ peak corresponds to C=C stretching in the aromatic benzene ring, a defining feature of the polymer backbone. Additional peaks at 1282 cm^−1^ (C-H bending and C-O stretching), 1174 cm^−1^ (C-H in-plane bending), and 859 cm^−1^ (C-C stretching in the benzene ring) further validate the PETG structure. The presence of a ring deformation mode at 632 cm^−1^ supports the aromatic character of the material. On the other hand, Hap is identified by its prominent peaks at 775 cm^−1^ and 997 cm^−1^, which correspond to phosphate group vibrations, particularly PO_4_^3−^ bending and symmetric stretching modes. The amount of folic acid in the sample is below the detection limit, while the characteristic Raman shifts of CS are masked by the dominant Raman signals of PETG, making their identification challenging in the spectrum. These spectral characteristics align with the findings from the literature, where Raman spectroscopy has been extensively applied to analyze PET-G in 3D printing, biomedical applications, and polymer recycling [62,63,64].

### 3.2. Biological Evaluation

In tissue engineering, it is crucial to test the compatibility of newly developed materials in a laboratory setting to ensure that they promote cell growth without compromising cell structure.

In this study, the biocompatibility of the newly developed porous 3D cubes was evaluated using the human preosteoblast cell line hFOB 1.19 as an in vitro cellular model. After a 24 h period of cell–material interaction, a series of biological tests were conducted to assess cell viability, distribution within the 3D structure of the porous cubes, and any potential cytotoxic effects.

**Figure 13:**
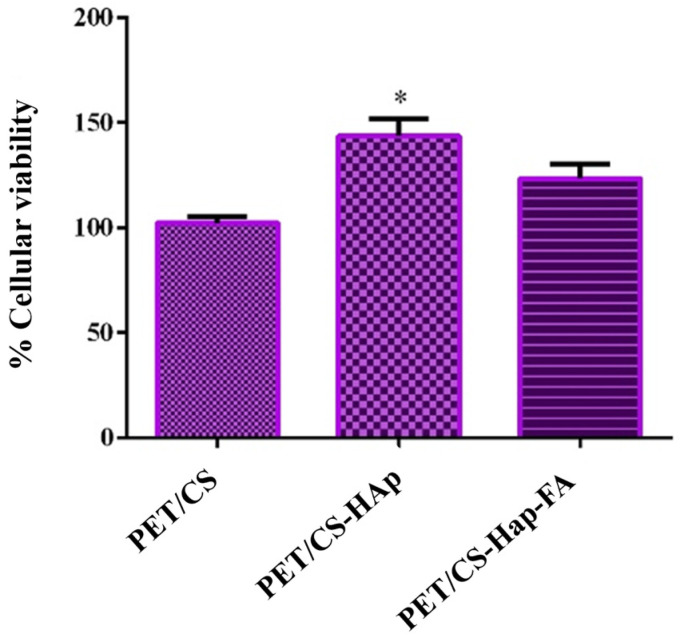
Graphical representation of the cellular viability of human preosteoblasts hFOB 1.19 after 24 h of contact with 3D porous cubes PETG/CS, PETG/CS-HAp, and PETG/CS-Hap-FA (* *p* ≤ 0.05).

The data obtained from the quantitative MTT test enabled the determination of human preosteoblast viability following 24 h of contact with the 3D porous cubes (Figure 13).

The Live/Dead test is a cell viability assay that allows for the simultaneous detection of live and dead cells using calcein and ethidium bromide (BrEth) staining. This analysis assesses specific parameters of cellular viability, including intracellular esterase activity and plasma membrane integrity. The test utilizes two fluorescent components to visualize the cells effectively: (i) calcein AM is a membrane-permeable live-cell labeling dye. Upon entering the cell, intracellular esterases cleave the acetoxymethyl (AM) ester group, yielding the membrane-impermeable calcein fluorescent dye that emits a bright-green fluorescence. Apoptotic and dead cells with compromised cell membranes do not retain calcein; (ii) BrEth is a compound that selectively enters cells with a damaged plasma membrane. Inside these cells, it binds to nucleic acids and generates a strong red fluorescence.

**Figure 14:**
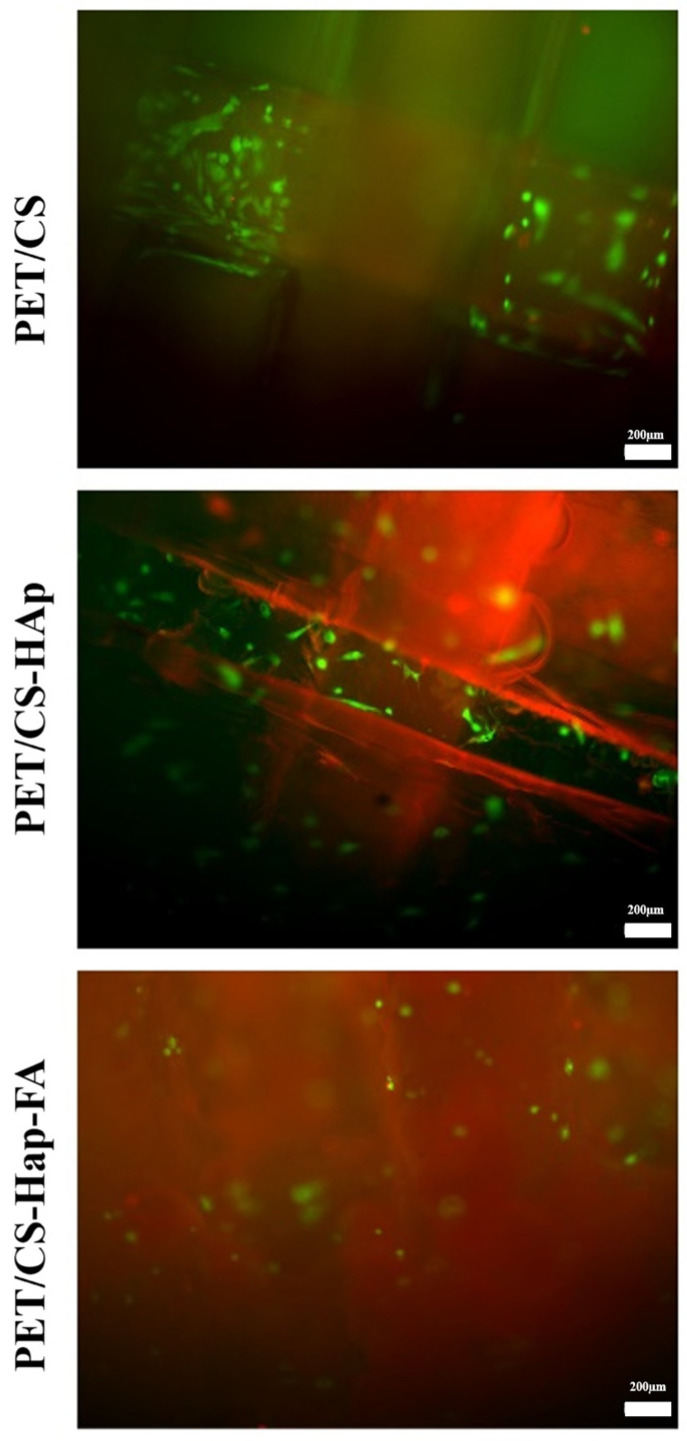
Fluorescence microscopy images highlighting live cells (green) and dead cells (red) after 24 h of contact with 3D porous cubes PETG/CS, PETG/CS-HAp, and PETG/CS-Hap-FA (magnification 10×).

The results demonstrate that the modified 3D porous cubes (PETG/CS-HAp and PETG/CS-Hap-FA) significantly enhance cell viability compared to the PET/CS control. Notably, the 3D porous cubes made from PETG/CS-HAp exhibited a marked improvement in cell viability relative to the control group. The Live/Dead test results corroborate the findings of the quantitative MTT test, revealing a substantial presence of brightly colored green viable cells across all three compositions. The modified 3D porous cubes (PETG/CS-HAp and PETG/CS-Hap-FA) facilitated greater cell penetration into the 3D structure of the materials. However, this was different in the case of the PETG/CS-Hap-FA, because the cells within the material structure cannot be fully visualized due to the autofluorescence of the material (Figure 14).

**Figure 15:**
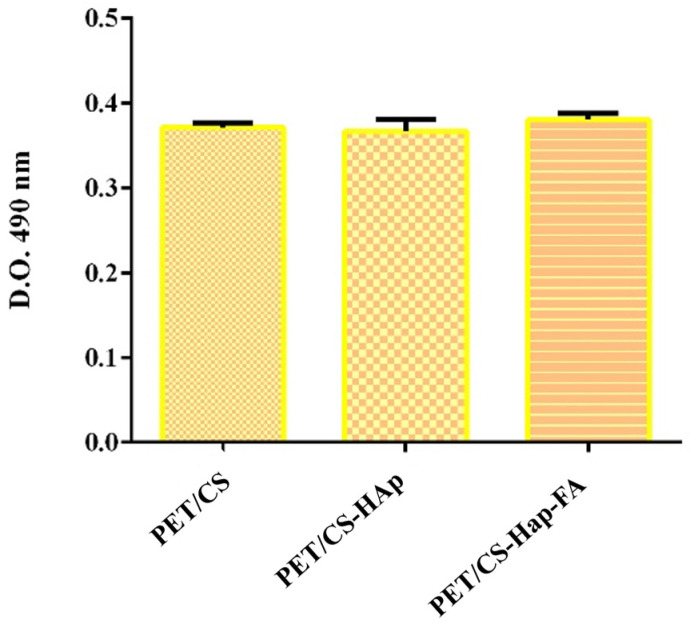
Graphical representation of the cytotoxicity of 3D porous cubes PETG/CS, PETG/CS-HAp, and PETG/CS-Hap-FA after 24 h of contact with human preosteoblasts.

An assessment was conducted to measure LDH levels to evaluate the potential cytotoxic effects of the 3D porous cubes made from PET material. LDH is an enzyme present within cells, and its release into the culture medium indicates damage to the cellular membrane, making it a valuable marker for assessing cytotoxicity. The results (Figure 15) demonstra that LDH levels remained consistent in the modified 3D porous cubes (PETG/CS-HAp and PETG/CS-Hap-FA) compared to the experimental control, with no significant changes identified. Thus, the findings suggest that the newly developed 3D porous cubes do not harm the selected in vitro cell model.

**Figure 16:**
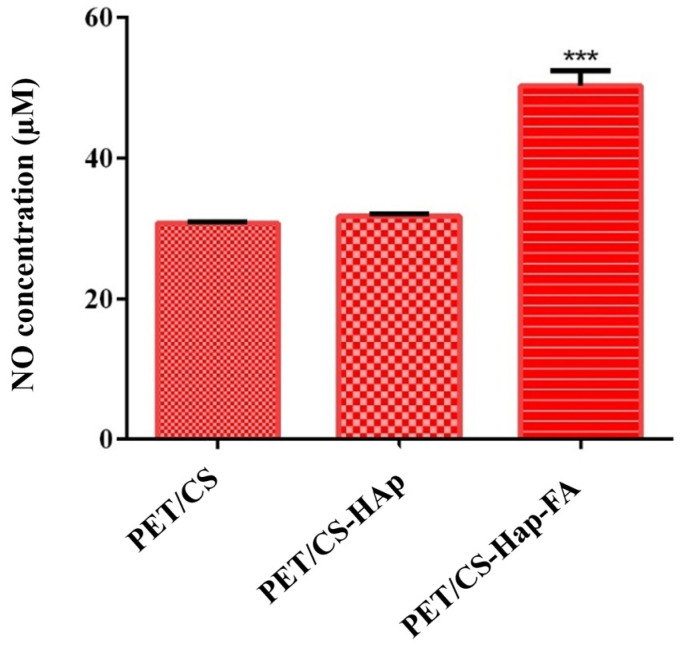
Graphical representation of the concentration of NO released into the culture medium by human preosteoblasts after 24 h of contact with the 3D porous cubes PET/CS, PET/CS-HAp, and PET/CS-Hap-FA (*** *p* ≤ 0.001).

Ultimately, the amount of nitric oxide (NO) was measured to determine the compatibility of the tested materials and assess any potential inflammatory effects they may have. The results obtained (Figure 16) show that the amount of NO released into the culture medium after 24 h of contact with human preosteoblasts hFOB 1.19 and the 3D PETG/CS-HAp scaffolds is comparable to that found in the experimental control. There were no significant changes in the concentration of NO observed. On the other hand, when examining culture medium samples taken 24 h after human preosteoblasts hFOB 1.19 were exposed to the 3D porous cubes PETG/CS-Hap-FA, a noticeable rise in the concentration of NO released into the culture medium was observed, surpassing that of the experimental control. However, the 1.6-fold increase in the concentration of NO in the 3D porous cubes of PETG/CS-Hap-FA does not automatically suggest that this material composition is cytotoxic. This is because NO is a molecule that has both positive and negative effects, depending on its concentration. Based on the impressive findings regarding the compatibility of the previously introduced 3D porous PETG/CS-Hap-FA cubes, it can be inferred that the notable rise in NO levels is not a sign of the material being harmful to cells. Instead, this increase can be attributed to the inclusion of folic acid in the material’s composition, which may promote NO production through its involvement in the synthesis of the enzyme nitric oxide synthase [65], its anti-inflammatory properties [66], and its ability to stimulate cellular metabolism [67]. Considering the given information, it can be inferred that the rise in NO levels in the culture medium of human preosteoblasts hFOB 1.19 cultivated on 3D porous PETG/CS-Hap-FA cubes does not surpass the concentration limit where NO becomes detrimental. Instead, the higher NO levels might play a role in facilitating cellular proliferation and differentiation, suggesting that increased NO levels are linked to the enhancement of preosteoblast differentiation [68].

The results indicate that the incorporation of hydroxyapatite and folic acid into the composition of 3D porous PETG/CS cubes yields a material with outstanding biological properties. This material enhances cell viability and preserves the morphology of preosteoblasts without causing harm to them. Both formulations demonstrated promising biocompatibility during in vitro testing. However, additional studies are necessary to confirm their compatibility with blood and their potential to promote bone growth.

## 4. Discussion

The modern approach to addressing medical challenges involves the integration of advanced technologies, while also prioritizing the use of natural materials or valorized waste whenever possible. Additionally, ensuring economic accessibility for a diverse range of patients is a key consideration. In this context, the present study explores the fabrication of porous scaffolds using 3D printing technology, with PETG as the base material. This porous structure, well-suited for bone applications, is further improved with hydroxyapatite (HAp), known for its osteoregenerative properties, and folic acid (FA), whose deficiency has been linked to bone fractures and other skeletal disorders. Furthermore, numerous studies in the scientific literature have investigated and tested materials similar to those developed in this study, reinforcing the relevance and potential of this approach in bone tissue engineering. Y. Hu et al. [69] prepared biomimetic hybrid organic–inorganic scaffolds composed of nano-hydroxyapatite, chitosan, chondroitin sulphate, and hyaluronic acid by combining biomimetic in situ synthesis with freeze-drying technology. The prepared scaffolds showed good mechanical properties and bioactivity as well as low cytotoxicity against osteoblasts as demonstrated by the MTT and alkaline phosphatase activity tests. Y. Zhang et al. reported a novel biomimetic composite nanofiber made from a hybrid CS/HAp material with a 30 wt% HAp mass ratio. This nanofiber was fabricated using a combination of in situ co-precipitation synthesis and an electrospinning process [70]. The authors proved that the nanocomposite material with the indicated composition could be successfully electrospun into nanofibers from an aqueous acetic acid solution while preserving the integrity of the HAp crystallites. Furthermore, biological evaluations of the nanofibers conducted on hFOB cells indicated that the excellent osteoconductivity of HAp stimulated the bone-forming capacity. Cell proliferation, mineral deposition, and morphology observed in the presence of the hybrid nanofibers compared to the plain CS scaffold further supported these findings. H. Wang et al. fabricated a periostin-loaded HAp/CS composite scaffold [22,71]. The scaffold was obtained without using any cross-linker. The periostin-loaded scaffold enhanced osteointegration and accelerated bone regeneration in a rat mandibular defect model. M.A. Nazeer et al. [72] prepared intercalated CS/HAp nanocomposites and nanoporous 3D scaffolds containing HAp, β-TCP, and calcium pyrophosphate. HAp nanoparticles were synthesized by the sol–gel method and further used in the preparation of CS/HAp nanocomposites by solution casting. Pure formic acid was used as a solvent resulting in the excellent homogenous dispersion of HAp nanoparticles in CS solutions. The thermal degradation of CS/HAp nanocomposites yielded layered 3D porous structures consisting of hydroxyapatite, tricalcium phosphate, and calcium pyrophosphate phases. C. Santos et al. prepared HAp nanoparticles loaded with folic acid and demonstrated their ability to induce osteoblastic differentiation [73]. W. Shi et al. developed an ingenious and simple method to directly immobilize FA on the surface of HAp nanoparticles without using a mediative reagent, such as a silane coupling agent [74]. The referenced studies highlight the extensive use of Hap and CS in bone tissue applications, as these materials are widely recognized for their versatile and beneficial properties in promoting bone regeneration and repair. The regular arrangement of calcium and phosphorus atoms in HAp nanoparticles leads to the formation of two distinct crystal planes. One of these, designated as the *c* plane, carries a negative charge and is predominantly composed of phosphate (PO_4_^3−^ ions referred to as P-sites) and hydroxide (OH^−^) ions. In contrast, the other plane, known as the *m* plane, is positively charged due to the abundance of calcium (Ca^2+^ denoted as C-sites) ions. Organic anions derived from pH-dependent ionizable compounds, such as folic acid, can facilitate P-site substitution by filling the vacancies left by the desorption of phosphate ions. This enables them to interact electrostatically with calcium ions. On the other hand, the Ca^2+^ ions on the *m*-plane surface (C-sites) can directly interact with the negatively charged organic ions, resulting in C-site coordination. This phenomenon paves the way for theranostic applications, as simultaneous P-site substitution and C-site coordination in a HAp nanoparticle could allow for the immobilization of two molecules with therapeutic and diagnostic functions on the same nanoparticle carrier. Polyethylene terephthalate (PET) has been widely utilized in various medical applications due to its biocompatibility, high uniformity, mechanical strength, and resistance to chemicals and abrasion. These properties make it an ideal material for the development of bone scaffolds, prosthetic vascular grafts, wound dressings, and other biomedical devices [75,76,77]. However, the main drawback of PET is associated with its high crystallinity resulting in significant printability limitations. It is possible to overcome this inconvenience by a glycol modification process using cyclohexanedimethanol and producing polyethylene terephthalate glycol (PETG) [78,79,80,81]. S. Sughanthy and colleagues conducted a dynamic mechanical analysis of PET/HAp biocomposites by monitoring changes in the storage modulus, loss modulus, and damping factor as the temperature increased. They concluded that the investigated material demonstrated favorable mechanical properties [77]. On the other hand, observing cell morphology by SEM, J. Jiang et al. showed that PET-HAp scaffolds have good biocompatibility with mouse fibroblast cells in vitro promoting cell differentiation and proliferation [82]. Applying the dip-coating method, Li et al. successfully modified the surface of a PET-manufactured LARS (Ligament Augmentation Reinforcement System; Surgical Implants and Devices) ligament with HAp. The modified HAp-PET ligament promoted graft-bone healing in a rabbit extra-articular model [30,83]. S. Sreeja et al. [84] synthesized a dual-functional scaffold consisting of a PET fibrous matrix that was surface-modified by phosphorylation and subsequently coated with an outer layer of biodegradable poly(hydroxyethyl methacrylate) polymer loaded with the antibiotic ciprofloxacin. This fabricated scaffold, designed for bone regeneration in cases of bacterial infections, such as osteomyelitis, demonstrated both osteoinductive properties and antimicrobial activity in vitro, effectively inhibiting *Staphylococcus aureus*. Ulf Tilman Strähle et al. [85] developed a similar cubic porous scaffold using the 3D printing technique, employing PETG as the printing material for the control sample. Additionally, they coated the PETG filament with nano-hydroxyapatite using a specialized coating machine. The SEM micrographs obtained for both the control PETG filaments and the hydroxyapatite-coated PETG filaments reveal similarities in the hydroxyapatite distribution within the PETG substrate, aligning with our findings. Furthermore, the EDS quantitative analysis from their study reported a phosphorus mass fraction ranging from 0.71% to 1.31% and a calcium mass fraction between 1.41% and 2.65%. In comparison, our results demonstrate a higher mass fraction of hydroxyapatite-related elements, with phosphorus reaching 2.84% and calcium 5.50% in the PETG/CS-HAp sample. This suggests that the immersion of the porous cubic PETG scaffold in the chitosan–hydroxyapatite mixture enhances the incorporation of hydroxyapatite nanoparticles, leading to a greater presence of bioactive material within the scaffold structure.

PETG has been studied and investigated as a material for bone scaffolds obtained through the 3D printing technique due to its favorable mechanical characteristics in terms of biocompatibility considering the interaction with the human environment, as well as the optimal characteristics related to the printing process, but also due to the mechanical properties that are a necessary characteristic in the development of scaffolds for hard tissues. Although PETG does not naturally have osteoconductive properties, like hydroxyapatite, its durability, flexibility, and impact resistance make it a suitable material for scaffolds intended for sustained bone regeneration and at the same time it can be modified in the sense of offering several activities necessary to stimulate bone regeneration. Moreover, due to the advanced technique of developing these scaffolds through 3D printing, the printing parameters can be varied so that the scaffold can be optimized in the sense of obtaining superior mechanical properties and implicitly an improved medical outcome. Numerous data in the literature have focused on varying the printing parameters of PETG scaffolds by modifying the layer thickness, infill density, temperature, and time of printing, and subsequently the mechanical properties of the materials obtained by varying the parameters were tested, thus making the correlations between the printing parameters and the final mechanical properties. A study by K. Durgashyam et al. [86] presents the obtaining of three PETG materials by varying the layer thickness (0.17, 0.23, 0.3 mm), feed rate (30, 40, 50 mm/s), and infill density (60, 70, 80 %), and testing the influence of these parameters on the tensile and flexural strength. Following the results, it was observed that layer thickness is the most important factor affecting tensile strength (according to the statistics carried out in that study, a percentage of 57.82% contribution was reached) by demonstrating the correlation that the thinner layer indicates a higher tensile strength in comparison with the thicker layer of 0.30 mm. At the same time, another important factor is the infill density, which participates positively in the increase in tensile strength, so that the highest value (80%) indicates the highest strength. Also, the third variable parameter, namely the feed rate, does not seem to have a significant impact on the mechanical properties, but the best result was obtained for the value of 40 mm/s. As for the flexural strength, in this case, the layer thickness also contributes the most to the result of the evaluation of the mechanical property, so that the more the layer thickness increases, the more the flexural strength decreases. The optimal feed rate was 40 mm/s, and the highest infill density had the best results correlated with the flexural strength.

In another study conducted by R. Srinivasan et al. [87], the effect of varying the infill density on the tensile strength of PETG-printed materials was investigated, ranging from 20% to 100%. The results show a clear correlation between the infill density and tensile strength, with 100% infill achieving the highest value of 32.12 MPa, significantly outperforming the 17.38 MPa recorded for 20% infill. Lakshmann Sri S. et al. [88] examined the mechanical properties of 3D-printed materials composed of PETG and polyamide by varying parameters such as the layer height, infill density, printing speed, bed temperature, and nozzle temperature. They found that the optimal settings for PETG, which yielded the best tensile strength, were as follows: nozzle temperature at 250 °C, bed temperature at 80 °C, printing speed at 40 mm/s, layer height at 0.2 mm, and infill density at 90%. Additionally, their findings indicate that compressive strength also improves with a higher infill density and lower layer height. Alfredo Ronca et al. [89] conducted tests comparing the mechanical properties of two types of PLA and PETG materials specifically for 3D-printed scoliosis back braces. Their results demonstrate that PETG exhibits superior mechanical properties, including tensile strength, impact resistance, and elongation at break, when compared to PLA.

In this study, PETG was utilized as a platform-type scaffold for bone regeneration, with printing parameters optimized based on the best results reported in the existing literature. These studies have demonstrated a strong correlation between the chosen printing parameters and the resultant mechanical properties of the materials (see Table 1). Consequently, PETG was ranked highly not only for its material properties, but also for the carefully selected printing parameters that were established to achieve favorable mechanical outcomes.

In our study, we fabricated by 3D printing three types of scaffolds intended for BTE applications: PETG-CS, PETG-CS-Hap, and PETG-CS-HAp-FA. The PETG-CS-printed biocomposite material was designed as the primary structural and tissue conductive component of the scaffold, benefiting from enhanced biocompatibility due to CS integration [79], while HAp provides a biomimetic surface for cell culture and ingrowth due to its excellent osteoconductive and osteoinductive capabilities. FA was introduced to the composition since several studies reported that vitamin B9 deficiency is associated with decreased bone quality and increased fracture risk [90].

## 5. Conclusions

This study focused on the fabrication and characterization of PETG-based porous scaffolds functionalized with CS, HAp, and FA for potential applications in bone tissue engineering. The scaffolds were successfully produced using 3D printing technology, providing a well-defined porous structure. The incorporation of bioactive components aimed to enhance biocompatibility and osteoregenerative potential, considering the critical role of HAp in bone regeneration and the association of FA deficiency with bone-related disorders.

The physicochemical characterization confirmed the structural and compositional integrity of the scaffolds. SEM and EDS analyses demonstrated the successful integration of HAp and FA, with elemental mapping highlighting their distribution across the scaffold’s surface and pores. FTIR analysis, although dominated by PETG signals, provided further evidence of functional group interactions. Raman results support the information obtained for the porous scaffolds.

The biological evaluation confirmed the cytocompatibility of the scaffolds through multiple assays. Cell viability tests demonstrated that the scaffolds did not exhibit cytotoxic effects, while Live/Dead assays provided additional confirmation of cell attachment and survival. Furthermore, LDH release measurements indicated no significant membrane damage, reinforcing the biocompatibility of the materials. Additionally, NO concentration assessment suggested a controlled inflammatory response, further supporting the cell-friendly nature of the scaffolds. However, while these tests confirm biocompatibility, they do not directly assess the bone regeneration potential of the scaffolds.

To fully validate the osteogenic capabilities of these scaffolds, future studies should focus on osteogenic differentiation and mineralization assays, such as Alizarin Red staining, ALP activity, and qPCR analysis of mineralization markers, to evaluate bone formation. Additionally, the stability of the HAp coating should be further investigated to ensure long-term bioactivity and mechanical integrity. Despite these limitations, the results highlight the promising potential of PETG-CS-HAp-FA scaffolds in bone tissue engineering, paving the way for further investigations, including in vivo studies, to explore their therapeutic applications.

## Figures and Tables

**Table 1:** Correlation between printing parameters and mechanical properties for PETG. Symbol: ↑—higher values, ↓—lower values. Created based on info available from [86,87,88,89].

Printing Parameter	Improved Effect on Tensile Strength	Improved Effect on Elastic Modulus	Improved Effect on Compressive Strength	Improved Effect on Impact Strength
Layer Height (mm)	↓	↓	↓	↓
Infill Density (%)	↑	↑	↑	↑
Printing Speed (mm/s)	↓	↓	↓	↓
Bed Temperature (°C)	↑	↑	↑	↑
Nozzle Temperature (°C)	↑	↑	↑	↑

## Data Availability

The original contributions presented in this study are included in the article. Further inquiries can be directed to the corresponding authors.
